# Perception of spousal involvement in breastfeeding among women attending infant welfare clinic in a private Tertiary Health Institution in Ogun State, Nigeria

**DOI:** 10.4314/ahs.v24i2.34

**Published:** 2024-06

**Authors:** Kolawole Sodeinde, Olufunmilola Abolurin, Olaitan Adeyoola, Idongesit Ekpo, Ashley Eto-Ihekwaba, Anuoluwapo Mabogunje, Ginikachukwu Ogbuehi, Ifeanyichukwu Ogbuiyi-chima, Tolulope Ogunsanya

**Affiliations:** 1 Department of Community Medicine, Babcock University, Ilisan, Ogun State, Nigeria; 2 Department of Paediatrics, Babcock University, Ilisan, Ogun State, Nigeria; 3 Department of Paediatrics, Lagos State University Teaching Hospital, Ikeja, Lagos State, Nigeria; 4 Department of Obstetrics and Gynaecology, Lagos State University Teaching Hospital, Ikeja, Lagos State, Nigeria

**Keywords:** Spousal involvement, breastfeeding, infant welfare clinic, Ogun State, Nigeria

## Abstract

**Background:**

Men's support improve breastfeeding practices. However, male involvement in breastfeeding practices is low, particularly in middle-and-low-income countries.

**Objective:**

This study assessed the perception of spousal involvement in breastfeeding among women attending infant welfare clinic in a private tertiary hospital in Ogun State, Nigeria.

**Methods:**

This descriptive cross-sectional research was conducted among 330 mothers. Data were collected using structured interviewer-administered questionnaire and analyzed using SPSS Version 22. Spousal involvement was assessed through the report of engagements of their husbands in 9 different activities. Those who participated in at least 5 and fewer than 5 activities were regarded as good and poor involvements respectively. Data were summarized using mean and standard deviation. Logistic regression was used to assess factors associated with perceived male involvement. P < 0.05 was statistically significant.

**Results:**

Mean age of participants was 32.3±6.5 years. Most (88.2%) of them reported that their husbands were involved in decision-making concerning breastfeeding. Women with monogamous relationships, who had tertiary education, and who were Christians were more likely to report good spousal involvement in breastfeeding.

**Conclusion:**

Educated women in monogamous relationships were better supported by their spouses. There is need for women's education and general empowerment to ensure better breastfeeding practices.

## Introduction

Breast milk contains growth factors, cytokines and hormones^1^ and is key in providing adequate nutrition for the proper growth and development of the infant.^2^ Exclusive breastfeeding (EBF) for a minimum of six months helps the child's immunity and survival and offers more benefit as compared to infant formula or cow milk.^2^ Such benefits include reducing the occurrence of allergies among infants who are at risk, 3 reducing morbidities and mortalities from diarrhea and acute respiratory infections.^3^ Since these are among the commonest childhood illnesses, breastfeeding, therefore, reduces the cost of medical care in the family. Breastfeeding an infant has also been shown to be associated with decreased occurrence of childhood and adolescent obesity and also reduced hypertension and dyslipidemia in adult life.^4^ Adequately breastfeeding babies also offer some advantages for mothers. It helps the woman to return to the pre-pregnancy weight, reduces the risk of breast and ovarian cancers, and reduces the chances of having osteoporosis and hip fractures in later years. Breastfeeding also improves mother-child bonding.^5^

Despite the importance and benefits of EBF, the practice has been shown to be particularly low in Africa, reported at a prevalence of 37%.^6^ In southwest Nigeria, only about one out of two newborns are exclusively breastfed.^7^ Breastfeeding practice is affected by several socio-cultural factors. For instance, interference from nuclear and extended families has been shown to affect breastfeeding practices in developing climes.^8^ According to various researchers in different parts of the world, men have a crucial role to play in the breastfeeding of their babies, and women who have the support of their husbands tend to breastfeed better and longer.^1^, ^8^, ^9^ Moreover, breastfeeding programmes involving both parents tend to have more productive outcomes as compared to those involving the woman alone.^10^, ^11^ However, male awareness and involvement in breastfeeding is poor in many low and middle income countries and this constitutes a major threat to satisfactory breastfeeding practices thus increasing the likelihood of ill-health and death among infants.^5^

Several factors have contributed to this suboptimal engagement of men in breastfeeding practices in many developing communities. These involved the patriarchal nature of these communities which accord much power and authority to the man^12^ Moreover, women are by default targeted for information on breastfeeding while the men are often sidelined.^13^ Also, the economic hardships in many developing nations make men to work extra hours away from home and hence have little or no time to contribute towards the health of their family including breastfeeding.^13^

There is a paucity of studies in developing countries on engaging men in promoting and supporting healthy breastfeeding practices and most of these studies have only been conducted in high-income countries.^14^ Moreover, various but contradictory effects of male participation in maternal and child health programmes relating to breastfeeding practices have been reported^14^ and there is, therefore, a need for further studies on this area of spousal engagement in countries like Nigeria.

This study assessed the perceived role of the male partners in breastfeeding practices among women accessing-services in the infant welfare clinic of a private tertiary hospital in Ogun State, southwest, Nigeria.

## Methods

### Study Area, Study Design, Study Population, and Sample Size Estimation

This was a facility-based cross-sectional study conducted between January and May 2022 at Babcock University Teaching Hospital (BUTH), Ogun State, Nigeria. BUTH, formerly known as Adventist Medical Centre is a 140-bedded tertiary hospital owned by the Seventh-day Adventist church in Nigeria. The hospital is situated at Ilisan equidistant between Ibadan and Lagos, the commercial capital of Nigeria.^15^ The hospital is made up of several departments that offer specialized care. However, the Community Medicine Department offers the primary level of care including immunization services which is provided in the Infant Welfare Clinic where this study was conducted.

The study participants were nursing mothers accessing childhood immunization services in the clinic. Women who were currently married and who were living with their spouses were included while women with their first child less than 6 months, women who for one reason or the other could not breastfeed their babies, or whose husband had some medical condition e.g. severe stroke that would prevent such men from supporting their wives were excluded.

The minimum sample size was determined using the standard formula for calculating the sample sizes of descriptive studies. A standard normal deviation of 1.96, a prevalence of 74.4% being the proportion of men that were involved in breastfeeding practices in Southern Ethiopia^16^ and a margin of error of 5% were inputted into the formula and this gave a minimum sample size of 292.67. Correcting for a non-response rate of 10%, the final calculation became 325.19. However, a total of 330 women participated in the study

### Sampling Method, Data Collection Tools, and Techniques, Study Measures

The purposive sampling method was used to select the study participants. Participants were recruited consecutively during routine clinic visits until the desired sample size was attained. A structured interviewer-administered questionnaire was used to elicit data with the help of trained research assistants. The instrument was constructed from a review of previous similar studies.^17^-^19^ The questionnaire sought information about the participants and their spouses' socio-demographic characteristics and the involvement of their spouses in breastfeeding practices. Data were elicited in the infant welfare clinic.

### Data management and analysis

The questionnaires were crosschecked for errors and cleaned. Data were entered into Statistical Package for Social Sciences (SPSS) software version 22 for analysis. Composite variables (aggregate scores) for spousal involvement in breastfeeding were computed from items on the questionnaire. A total of nine items were assessed giving a total score of 9. Anyone whose spouse participated in a minimum of 5 out of 9 activities was considered to have good involvement while those whose spouse participated in 4 or fewer activities were considered to have poor spousal involvement in breastfeeding. The analyzed data were presented as frequency tables. Data summary was done using mean, standard deviation, and proportions. Chi-squares were used to test for associations between spousal involvement in breastfeeding and other categorical variables. Logistic regression was used to assess factors associated with perceived male involvement in breastfeeding. The level of significance was set at 5%

### Ethical consideration

Ethical approval was obtained from the Babcock University Health Research and Ethics Committee (BUHREC 538/20). Verbal and written informed consent was obtained from each participant. Strict confidentiality was maintained throughout the study which included ensuring auditory privacy during data collection in the infant welfare clinic.

## Results

Table 1 shows that the mean age of the participants was 32.3±6.5 years while the mean age of their spouses was 39.4± 8.1. Most (91.2%) of the participants were in monogamous marriages. A higher proportion (55.2%) of the participants had one or two children while the remaining 44.8% had more than two children. The majority (70%) of the participants had tertiary education, one-quarter (25.5%) had secondary education while a few (0.9%) had no formal education. Most of the participants (84.5%) and their spouses (97.6%) were employed. One-fifth (19.4%) of the participants had their spouses earning less than one hundred thousand naira (about $217) in a month while about one-third (31.5%) of the participants had their spouses earning between one hundred and three hundred thousand naira monthly (about $217-$651)

**Table 1:** Socio-demographic characteristics (N=330)

Variable	Frequency	Percentage
**Age (Years)**		
≤24	19	5.8
25-34	222	67.3
35-44	67	20.3
≥45	22	6.6
**Type of Marriage**		
Monogamous	301	91.2
Polygamous	29	8.8
**Number of Children**		
≤2	182	55.2
>2	148	44.8
**Educational Status**		
No formal education	3	0.9
Primary	12	3.6
Secondary	84	25.5
Tertiary	231	70.0
**Occupation**		
Employed	279	84.5
Unemployed	51	15.5
**Ethnicity**		
Yoruba	209	63.3
Igbo	78	23.6
Hausa	13	3.9
Others	30	9.2
**Religion**		
Christianity	273	82.7
Islam	57	17.3
**Age of Spouse (Years)**		
21-30	38	11.5
31-40	174	52.7
41-50	87	26.4
51-60	24	7.3
≥61	7	2.1
**Occupation of Spouse**		
Employed	322	97.6
Unemployed	8	2.4
**Ethnicity of Spouse**		
Yoruba	220	66.7
Igbo	77	23.3
Hausa	12	3.6
Others	21	6.4
**Religion of Spouse**		
Christianity	264	80.0
Islam	65	19.7
Traditional Religion	1	0.3
**Spouse's Income (Naira)**		
<100,000	64	19.4
100,000-299,999	104	31.5
300,000-499.999	60	18.2
≥500,000	52	15.8
Don't know	50	15.1

Table 2 shows different ways the women mentioned that their husbands supported them concerning breastfeeding. Most (88.2%) reported that their husbands were involved in decision-making concerning breastfeeding their children. One-third (32.7%) of the participants reported that their husbands stayed at home more during the breastfeeding period while a little above one-third (36.7) reported that their husbands provided feeding cups to feed their infant with extracted breast milk in instances like work schedules when the mothers could not breastfeed their babies directly. About half (51.5%) of the women reported that their husbands supported them emotionally while close to three-fifths (57.6%) said their spouses took them and the children to the postnatal clinic. The lowest proportion of women (29.4) was those who reported that their husbands helped with house chores during the breastfeeding period.

**Table 2:** Perceived Spousal Involvement in Breastfeeding

Variable	Frequency	Percentage
**Spouse involvement in decisions related to breastfeeding**		
Yes	291	88.2
No	39	11.8
**Stay at home more during breastfeeding**		
Yes	108	32.7
No	222	67.3
**Provide feeding cup**		
Yes	121	36.7
No	209	63.3
**Financial Support**		
Yes	292	88.5
No	38	11.5
**Take Spouse and child to Post-natal clinic**		
Yes	190	57.6
No	140	42.4
**Emotional support to wife on breastfeeding**		
Yes	170	51.5
No	160	48.5
**Help with household chores during breastfeeding**		
Yes	97	29.4
No	233	70.6
**Frequency of spouses staying at home during breastfeeding**		
Most time of the day	134	40.6
At night	132	40.0
Weekly (weekends only)	64	19.4
**Accompanied to at least one ANC visit by the spouse**		
Yes	165	50.0
No	165	50.0
**Spousal Involvement Score**		
Good Involvement	219	66.4
Poor Involvement	111	33.6

Table 3 shows that the type of marriage (p=0.001), the woman's educational status (p=0.001), the woman's religion (p=0.002) the woman's ethnicity (p=0.046), and the religion of the spouse (p=0.046) were statistically significantly associated with spousal involvement in breastfeeding.

**Table 3:** Association between selected characteristics and perceived spousal involvement in breastfeeding

Variable	Spousal Involvement Category Poor involvement n (%)	Good involvement n(%)	Test Statistics
**Age**			
≤24	9 (47.4)	10 (52.6)	
25-34	69 (31.1)	153 (68.9)	
35-44	26 (38.8)	41 (61.2)	χ^2^= 3.089
≥45	7 (31.8)	15 (68.2)	p= 0.378
**Type of Marriage**			
Monogamous	93 (30.9)	208 (69.1)	χ^2^= 11.514
Polygamous	18 (62.1)	11 (37.9)	p= 0.001
**Number of Children**			
≤2	63 (34.6)	119 (65.4)	χ^2^= 0.174
>2	48 (32.4)	100 (67.6)	p= 0.676
**Educational status**			
No formal education	1 (33.3)	2 (66.7)	
Primary	8 (66.7)	4 (33.3)	
Secondary	38 (45.2)	46 (54.8)	χ^2^= 14.570*
Tertiary	64 (27.7)	167 (72.3)	p= 0.001
**Occupation**			
Employed	90 (32.3)	189 (67.7)	χ^2^= 1.536
Unemployed	21 (41.2)	30 (58.8)	p= 0.215
**Religion**			
Christianity	82 (30.0)	191 (70.0)	χ^2^= 9.175*
Islam	29 (50.9)	28 (49.1)	p=0.002
**Ethnicity**			
Yoruba	81 (38.8)	128 (61.2)	
Igbo	17 (21.8)	61 (78.2)	
Hausa	3 (23.1)	10 (76.9)	χ2= 8.004
Others	10 (33.3)	20 (66.7)	p= 0.046
**Age of Spouse**			
21-30	11 (28.9)	27 (71.1)	
31-40	58 (33.3	116 (66.7	
41-50	29 (33.3)	58 (66.7)	
51-60	11 (45.8)	13 (54.2)	χ^2^= 2.065
≥61	2 (28.6)	5 (71.4)	p= 0.724
**Religion of Spouse**			
Christianity	82 (31.1)	182 (68.9)	χ^2^= 3.923
Islam	29 (43.9)	37 (56.1)	p=0.048
**Ethnicity of Spouse**			
Yoruba	78 (35.5)	142 (64.5)	
Igbo	23 (29.9)	54 (70.1)	
Hausa	2 (16.7)	10 (83.3)	χ^2^= 2.550
Others	8 (38.1)	13 (61.9)	p= 0.466
**Occupation of Spouse**			
Employed	109 (33.9)	213 (66.1)	χ^2^= 0.274
Unemployed	2 (25.0)	6 (75.0)	p= 0.601
**Spouse Income**			
<100,000	18 (28.1)	46 (71.9)	
100,000-299,999	34 (32.7)	70 (67.3)	
300,000-499,999	21 (35.0)	39 (65.0)	
≥500,000	18 (34.6)	34 (65.4)	χ^2^= 1.892
Don't know	20 (40.0)	30 (60.0)	p= 0.756

Table 4 shows that women who were in a monogamous relationship were about 3 times more likely to report good spousal involvement in breastfeeding as compared to women in polygamous relationships (OR= 2.81; 95% C.I= 1.09-7.26). Also, women who were Christians were about 4 times more likely to report good spousal involvement as compared to those who practiced Islam (OR 3.85; 95% C.I=1.03-14.46). Women who had no formal education were also 48% less likely to report good spousal involvement in breastfeeding as compared to women with tertiary education (OR=0.52; 95% C.I= 0.04-6.29)

**Table 4:** Participant characteristics and multivariate relationship with perception of spousal involvement in breastfeeding

Variable	Odd's Ratio	95% interval	Confidence	p-value
**Type of Marriage**				
Monogamous	2.81	1.09-7.26		0.032
Polygamous	1.00			
**Educational Status**				
No formal education	0.52	0.04-6.29		0.607
Primary	0.20	0.06-0.73		0.015
Secondary	0.59	0.34-1.04		0.068
Tertiary	1.00			
**Religion**				
Christianity	3.85	1.03-14.46		0.046
Islam	1.00			
**Tribe**				
Yoruba	1.04	0.45-2.41		0.934
Hausa	3.73	0.70-19.86		0.123
Igbo	1.81	0.70-4.65		0.219
Others	1.00			
**Spouse Religion**				
Christianity	1.00			
Islam	0.35	0.10-1.29		0.115

## Discussion

Most of the participants reported that their spouses were involved in decision-making concerning breastfeeding while fewer participants reported the involvement of their spouses in other support activities such as staying at home more during breastfeeding, providing emotional support, and helping with house chores during breastfeeding. Women who were in a monogamous relationship, who were Christians, or who had tertiary education were more likely to report spousal involvement in breastfeeding.

Female education is important as it helps in empowering women in various ways. According to a study that evaluated the relational empowerment of women from 70 developing countries, a direct relationship was found between women's education and their opportunities within the household including improved decision-making opportunities, improved support, and reduced opposition from the spouse.^20^ These may result from better access of such women to breastfeeding-related information and high socio-economic status. Moreover, educated women are more likely to be married to educated men who may probably have more access to breastfeeding-related information from multiple sources. Little wonder our study observed that the women's level of education had positive relationships with perceived spousal support in breastfeeding. This finding is beneficial since spousal involvement, particularly in the early postpartum period, has been shown to improve breastfeeding intentions, activities, and duration while the failure of men to support their wives during this period is associated with undesirable breastfeeding experiences.^21^,^22^ Therefore, the direct relationship between female education and male involvement in breastfeeding observed in this study will lead to better breastfeeding activities. Furthermore, in a study conducted in Indonesia, Laksono opined that women's education has a direct association with breastfeeding.^23^

Our study revealed that women in monogamous marriages were more likely to receive support from their spouses during breastfeeding. This observation was similar to the findings from a Malian study where Bove reported that women who were in monogamous relationships were more likely to be accompanied to the health facility by their spouses. Such women were also more likely to pay for medical services when compared to those in polygamous relationships.^24^

Women's social relationships have been linked to different aspects of their health.^25^ For instance, studies have shown that the type of marriage influences various aspects of the reproductive health of women.^26^,^27^ In Ghana, Gyimah postulated that polygynous type of marriage may adversely affect child health including survival.^28^

Several reasons may be responsible for the increased support of monogamous men towards their wives during breastfeeding and by extension, other aspects of reproductive health. First polygamy may be said to divide the attention of the man and lay on him more burden in providing support to the various wives. Moreover, there may be other subtle factors like bias or preference of a particular partner especially if the preferred wife was not the one included in this study

Other socio-cultural characteristics such as religion and ethnicity were also noted to be associated with male involvement in breastfeeding in this current study. In agreement with this observation, studies in various parts of the world have also shown that cultural and religious factors modulate breastfeeding practices.^29^,^30^ In a related development, Reinsma posited that cultural beliefs may be incorporated in developing health education interventions so as to promote the practice of exclusive breastfeeding, especially in developing countries.^31^ A study by Wanjohi in Kenya noted that husbands teach their wives positive cultural beliefs to improve breastfeeding practices. On their own part, the women easily adopted these spouse-taught beliefs since they lent a voice to the possibility of better nutrition and improved health outcomes among adequately breastfed children.^32^

Observations from this study are expected to inform decisions and interventions that will promote male involvement in providing breastfeeding support for their spouses. This ultimately will lead to better mother and child health and curb many common childhood causes of morbidities and mortalities.

This study however has some limitations. First, the cross-sectional nature of the study did not allow a temporal assessment of the relationship between participants' characteristics and perceived spousal involvement in breastfeeding. Besides, asking the women about the involvement of their spouses in breastfeeding activities had attending recall bias. Future opportunities for research may include conducting mixed-method studies where qualitative research components will be used to explore perceptions of women about the involvement of their spouses in breastfeeding practices. Also, the studies may be conducted among men to assess their involvement in breastfeeding practices.

## Conclusion/Recommendation

Participants in this study had the perception that their spouses were more involved in decision-making as compared to other aspects of breastfeeding support such as giving emotional support or staying more at home during breastfeeding of children. Women who were in monogamous relationships, who were Christians, or who had tertiary education were more likely to report good involvement of their spouses in breastfeeding. The need for women's education and general empowerment cannot be over-emphasized. Couples in polygamous relationships also need to be educated and encouraged as regards joint effort in ensuring successful breastfeeding exercises for their children.

## References

[1] Mannion C, Hobbs A, McDonald S, Tough S (2013). Maternal perceptions of partner support during breastfeeding. Int Breastfeed J.

[2] Mulugeta WA, Netsanet HB, Nigusie BT, Selam FK (2017). Knowledge and Attitude towards Exclusive Breast Feeding among Mothers Attending Antenatal and Immunization Clinic at Dabat Health Center, Northwest Ethiopia: A Cross-Sectional Institution Based Study. Nurs Res Pract.

[3] Hossain S, Mihrshahi S (2022). Exclusive Breastfeeding and Childhood Morbidity: A Narrative Review. Int J Environ Res Public Health.

[4] World Health Organization (2023). Exclusive breastfeeding to reduce the risk of childhood overweight and obesity.

[5] Yourkavitch JM, Alvey JL, Prosnitz DM, Thomas JC (2017). Engaging men to promote and support exclusive breastfeeding: a descriptive review of 28 projects in 20 low- and middle-income countries from 2003 to 2013. J Health Popul Nutr.

[6] Stanaway J D (2018). Global, regional, and national comparative risk assessment of 84 behavioural, environmental and occupational, and metabolic risks or clusters of risks for 195 countries and territories, 1990–2017: a systematic analysis for the Global Burden of Disease Study 2017. Lancet.

[7] Adebayo AM, Ilesanmi OS, Falana DT, Olaniyan SO, Kareem AO, Amenkhienan IF (2021). Prevalence and predictors of exclusive breastfeeding among mothers in a semi-urban Nigerian community: a cross-sectional study. Ann Ib Postgrad Med.

[8] Mihrshahi S, Ichikawa N, Shuaib M, Oddy W, Ampon R, Dibley MJ (2015). Prevalence of exclusive breastfeeding in Bangladesh and its association with diarrhea and acute respiratory infection: results of the multiple indicator cluster survey 2013. J Health Popul Nutr.

[9] Kishore MS, Kumar P, Aggarwal AK (2012). Breastfeeding knowledge and practices amongst mothers in a rural population of North India: a community-based study. J Trop Pediatr.

[10] Özlüses E, Çelebioglu A (2014). Educating fathers to improve breastfeeding rates and paternal-infant attachment. Indian Pediatr.

[11] Li R, Darling N, Maurice E, Barker L, Grummer-Strawn LM (2015). Breastfeeding rates in the United States by characteristics of the child, mother, or family. Pediatrics.

[12] Agrawal J, Chakole S, Sachdev C (2022). The Role of Fathers in Promoting Exclusive Breastfeeding. Cureus.

[13] Bulemela J, Mapunda H, Snelgrove-Clarke E (2019). Supporting breastfeeding: Tanzanian men's knowledge and attitude towards exclusive breastfeeding. Int Breastfeed J.

[14] Ogbo FA, Eastwood J, Page A, Arora A, McKenzie A, Jalaludin B (2017). Prevalence and determinants of cessation of exclusive breastfeeding in the early postnatal period in Sydney, Australia. Int. Breastfeed. J.

[15] Shobowale E, Elikwu C, Olusanya AO, Abiodun A (2016). The Profile of Tuberculosis Infection at the Babcock University Teaching Hospital. IJMBR.

[16] Abera M, Abdullahi M, Wakayo T (2017). Fathers' Involvement In Breastfeeding Practices Ad Associated Factors Among Households Having Children Less Than Six Months In Southern Ethiopia: A Cross-Sectional Study. Pediatr Ther.

[17] Ogbo FA, Page A, Idoko J, Claudio F, Agho KE (2017). Have policy responses in Nigeria resulted in improvements in infant and young child feeding practices in Nigeria?. Int Breastfeed J.

[18] Kidane G, Oksana Z, Afrwork M, Danielle G (2021). A cross-sectional comparison of breastfeeding knowledge, attitudes, and perceived partners' support among expectant couples in Mekelle Ethiopia. Int Breastfeed J.

[19] Rollins NC, Bhandari N, Hajeebhoy N, Horton S, Lutter CK, Martines JC (2016). Why invest, and what it will take to improve breastfeeding practices?. The Lancet.

[20] Le K, Nguyen M (2021). “How Education Empowers Women in Developing Countries”. The B.E. Journal of Economic Analysis & Policy.

[21] Gözükara F (2014). Key Factor for Achievement of Breastfeeding: Providing Father Support and Roles of Nurses. Harran Üniversitesi Tıp Fakültesi Dergisi.

[22] Rempel LA, Rempel JK, Moore KC (2017). Relationships between types of father breastfeeding support and breastfeeding outcomes. Matern Child Nutr.

[23] Laksono AD, Wulandari RD, Ibad M, Kusrini I (2021). The effects of mother's education on achieving exclusive breastfeeding in Indonesia. BMC Public Health.

[24] Bove RM, Vala-Haynes E, Valeggia C (2014). Polygyny and women's health in rural Mali. J Biosoc Sci.

[25] Bedrov A, Gable SL (2023). Thriving together: the benefits of women's social ties for physical, psychological and relationship health. Phil. Trans. R. Soc.

[26] Odusina EK, Ayotunde T, Kunnuji M, Ononokpono DN, Bishwajit G, Yaya S (2020). Fertility preferences among couples in Nigeria: a cross-sectional study. Reprod Health.

[27] Fafard St-Germain AA, Kirby RS, Urquia ML (2022). Reproductive health among married and unmarried mothers aged less than 18, 18-19, and 20-24 years in the United States, 2014-2019: A population-based cross-sectional study. PLoS Med.

[28] Gyimah SO (2009). Polygynous marital structure and child survivorship in sub-Saharan Africa: some empirical evidence from Ghana. Soc Sci Med.

[29] Daud N, Nordin N, Shukry ASM, Ali RM, Mohamed Z (2014). A Study of the Understanding amongst Academia towards the Islamic Concept of Breastfeeding. Asian Soc Sci.

[30] Bernard JY, Rifas-Shiman SL, Cohen E, Lioret S, de Lauzon-Guillain B, Charles MA, Kramer MS, Oken E (2020). Maternal religion and breastfeeding intention and practice in the US Project Viva cohort. Birth.

[31] Reinsma K, Bolima N, Fonteh F, Okwen P, Yota D, Montgomery S (2012). Incorporating cultural beliefs in promoting exclusive breastfeeding. Afr J Midwifery Womens Health.

[32] Wanjohi M, Griffiths P, Wekesah F, Muriuki P, Muhia N, Musoke RN (2016). Sociocultural factors influencing breastfeeding practices in two slums in Nairobi, Kenya. Int Breastfeed J.

